# Pediatric Hypertension: A Case Report of an Unusual Presentation

**DOI:** 10.7759/cureus.91277

**Published:** 2025-08-30

**Authors:** Marta Baldo, Francisca Andrade, Ana Macedo

**Affiliations:** 1 Family Medicine, Unidade de Saúde Familiar (USF) Novo Sentido, Unidade Local de Saúde (ULS) São João, Porto, PRT

**Keywords:** family medicine, hypertension, pediatrics, renal dilatation, ureteropelvic junction obstruction

## Abstract

Pediatric hypertension is often asymptomatic but can signal underlying secondary causes such as renal or renovascular diseases. Ureteropelvic junction obstruction (UPJO) is a leading cause of upper urinary tract dilation in children. Although it typically presents with abdominal pain, hematuria, or urinary tract infections, it may, in rare cases, manifest solely as hypertension.

This case report describes an eight-year-old boy with asymptomatic hypertension and proteinuria detected during routine screening. Imaging revealed a left-sided UPJO with renal impairment, as well as horseshoe kidneys with fusion at the lower poles. The patient was treated with antihypertensives and underwent successful surgical correction, with ongoing nephrology follow-up.

This case underscores the importance of routine blood pressure monitoring in children to enable early diagnosis of silent renal pathologies, facilitating timely treatment and improved outcomes.

## Introduction

Hypertension is a significant contributor to global morbidity and mortality. The estimated prevalence of pediatric hypertension ranges from two to five percent, with a noted increase in recent decades largely attributed to rising childhood obesity rates [1,2]. No national prevalence data are currently available in Portugal.

In children, the presence of hypertension warrants a thorough evaluation. Between the ages of seven and 12, the most common secondary causes include parenchymal renal disease, renovascular anomalies, and endocrine dysfunction [1]. Among these, ureteropelvic junction obstruction (UPJO), a partial or intermittent blockage at the junction between the renal pelvis and ureter, is the leading cause of renal dilation in pediatrics. It is more prevalent in males and usually unilateral [3,4]. Often diagnosed prenatally through the detection of unilateral hydronephrosis on obstetric ultrasound, the diagnosis is confirmed postnatally with renal ultrasound and diuretic renogram. In infants, clinical presentation may include palpable abdominal mass, urinary tract infections, hematuria, or failure to thrive. In older children, it can present with intermittent abdominal pain, hematuria, kidney stones, acute pyelonephritis, renal injury, or, more rarely, isolated hypertension [4].

This case aims to emphasize the importance of regular monitoring of pediatric health parameters, particularly blood pressure, as a critical strategy for early detection of underlying pathology.

## Case presentation

This is the case of an eight-year-old boy with no significant personal or family medical history. He attended a routine eight-year health check at his local Family Health Unit, asymptomatic at the time. Physical examination revealed elevated blood pressure values above the 95th percentile (>P95) in both upper limbs and the left lower limb. There were no significant findings on auscultation or weight abnormalities. A detailed history did not reveal any related symptoms such as fever, vomiting, headaches, urinary complaints, or mood changes. A balanced diet and adequate fluid intake were reported. Dipstick urinalysis showed proteinuria. Previous health records confirmed normal blood pressure values during earlier well-child visits, with the most recent measurement taken at age six.

Ambulatory blood pressure monitoring (ABPM) confirmed grade I systolic-diastolic hypertension with a "dipper" pattern. Renal ultrasound demonstrated exuberant left-sided calyceal dilatation, likely congenital, with marked cortical thinning in an enlarged kidney. The right kidney showed no abnormalities. Laboratory and cardiac evaluations (ECG and echocardiogram) were unremarkable. The patient was referred to Pediatrics. During this consultation, the mother reported nocturnal enuresis and irregular urination with occasional daytime leakage. No complaints of pain or macroscopic hematuria were noted.

As part of the hospital assessment, further diagnostic imaging was performed, including renal Doppler ultrasonography and a MAG3 (mercaptoacetyltriglycine) diuretic renogram. Doppler ultrasonography revealed findings consistent with chronic obstructive nephropathy, including difficult visualization of the left renal artery and a mildly elevated resistance index. The MAG3 renogram demonstrated reduced function of the left hemikidney with pelvicalyceal stasis and suspected drainage obstruction, while the right hemikidney had non-obstructive pelvic stasis.

**Figure 1:**
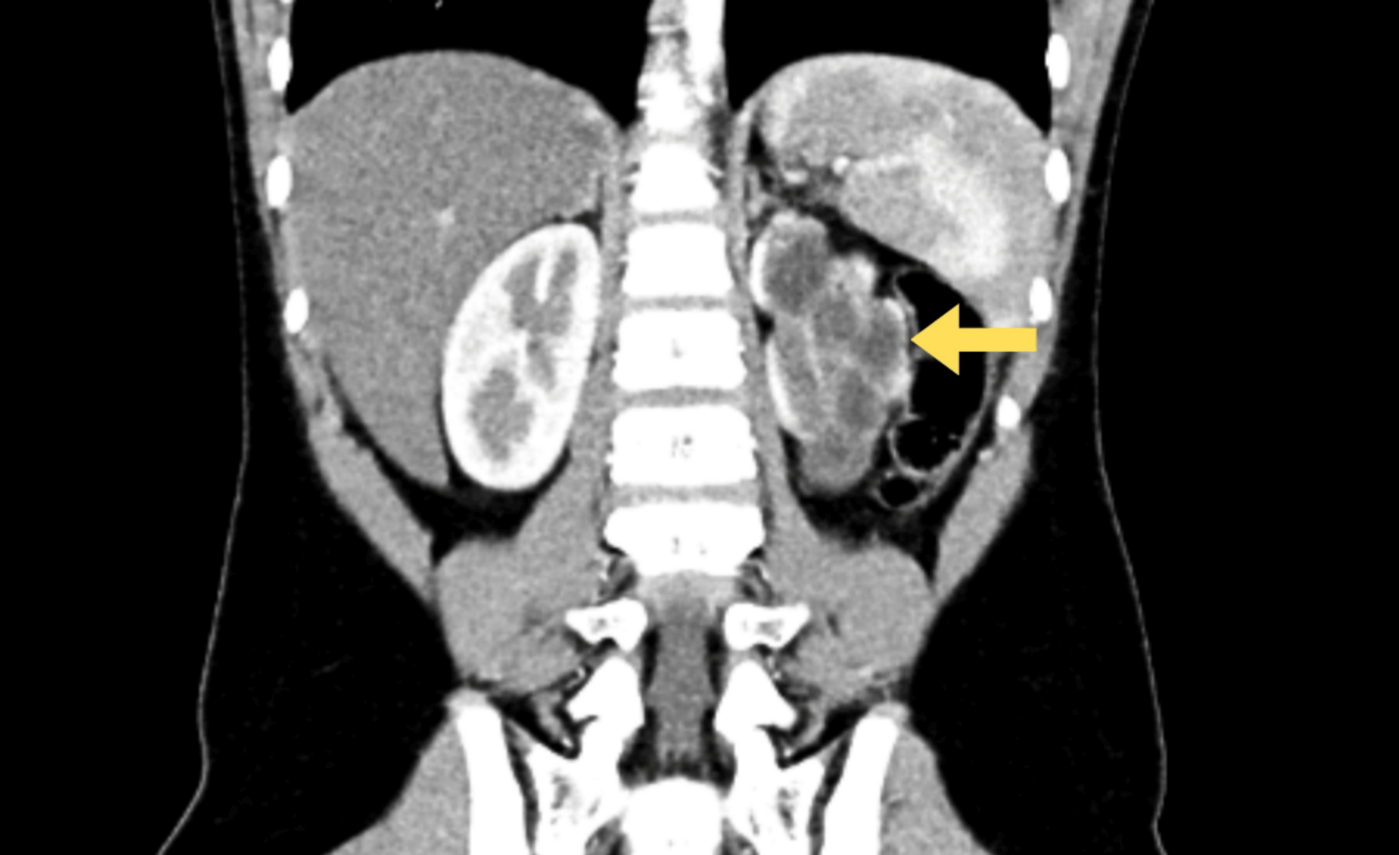
Reno-pelvic CT image revealing cortical thinning and calyceal ectasias of the left kidney.

The patient was started on lisinopril 5 mg daily and referred to Pediatric Surgery. A reno-pelvic CT scan was performed for better anatomical definition and revealed horseshoe kidneys with fusion at the lower poles, associated with diffuse left renal parenchymal thinning and multiple calyceal ectasias, suggestive of chronicity (Figure 1). The right renal parenchyma was preserved. There was no dilatation of the left ureter, consistent with UPJO. The right kidney showed no pelvicalyceal dilatation but a probable extra-sinusal pelvis (13 mm anteroposterior diameter) without calyceal involvement. No suspicious renal lesions were identified. Two main renal arteries were present on each side, with smaller caliber on the left and no evident stenosis. 

He underwent a left-sided Anderson-Hynes dismembered pyeloplasty in a horseshoe kidney without complications. He is currently under follow-up in Pediatric Nephrology, clinically stable, on lisinopril 7.5 mg daily, and without urinary symptoms. The current dosage was adjusted during follow-up due to persistently elevated blood pressure readings (>P95) observed both at home and during medical appointments, approximately 10 months after diagnosis. Since then, he has remained stable, with good treatment adherence and no further complications over nearly three years of follow-up.

## Discussion

Pediatric hypertension is often asymptomatic, which is why leading medical societies, including the American Academy of Pediatrics, recommend routine blood pressure screening for all children aged three years and older during health supervision visits [2].

The initial evaluation of hypertension in children aims to distinguish between primary and secondary causes, identify treatable conditions, assess early cardiovascular risk factors, and determine the need for pharmacological therapy [5]. A detailed medical history should explore symptoms suggestive of secondary causes, past medical history, use of medications or substances that may raise blood pressure, and a family history of cardiovascular or genetic disorders [6]. Physical examination must be thorough, looking for signs of specific diseases such as skin changes, edema, cardiac or abdominal murmurs, pulse or pressure discrepancies between limbs, endocrine abnormalities, or autoimmune manifestations [6]. Timely identification and management of hypertension are associated with improved long-term cardiovascular outcomes.

UPJO is the most common cause of upper urinary tract obstruction in children and may progress silently until advanced stages with significant renal injury. Its clinical presentation varies with age. Although rare, hypertension can be the initial manifestation of UPJO, resulting from activation of the renin-angiotensin system due to chronic obstruction [4]. This case illustrates such an atypical presentation, in which hypertension was the only initial clinical finding.

The diagnostic approach followed a stepwise strategy appropriate to the primary care setting. After elevated blood pressure was identified during a routine visit, complementary investigations were carried out to confirm the diagnosis and evaluate for secondary causes. ABPM confirmed hypertension and characterized the blood pressure profile. This tool is particularly useful in children, where in-office measurements can be less reliable, and helps differentiate true hypertension from “white-coat” hypertension [6,7]. It also provides relevant prognostic information, such as the dipper pattern, which is associated with lower cardiovascular risk [7]. Proteinuria and renal ultrasound findings suggested renal involvement, warranting referral to a specialist. In secondary care, Doppler ultrasound and a MAG3 renogram confirmed chronic obstructive nephropathy. Imaging also revealed horseshoe kidneys, a congenital anomaly that increases the risk of UPJO and adds complexity to anatomical assessment and surgical planning.

Despite the absence of symptoms at presentation, this case revealed significant structural renal changes suggestive of chronic kidney injury. The delayed diagnosis of UPJO, accompanied by marked left-sided renal hypofunction and cortical atrophy, underscores the importance of early and systematic blood pressure screening in children. Ureteropyeloplasty remains the gold standard treatment for UPJO and is effective in relieving obstruction and preserving renal function [8]. However, when substantial renal damage is already present at the time of diagnosis, the potential for functional recovery may be limited [3]. This emphasizes the critical need for early detection of silent obstructive uropathies to optimize long-term renal outcomes [3].

Beyond clinical and surgical management, the psychosocial impact of such a diagnosis on a child and family must be acknowledged. Transitioning from a state of apparent health to a condition requiring chronic therapy, specialized follow-up, and surgery can cause anxiety, fear, and adherence challenges. Emotional support and effective communication between healthcare teams, the child, and caregivers are essential to ensure adherence and favorable outcomes.

In this context, the role of the family physician is fundamental, not only in early detection but also in promoting health literacy. Empowering parents to recognize warning signs, understand the importance of regular follow-up, and adhere to medical advice is a key pillar in preventing chronic kidney disease and cardiovascular complications in adulthood.

## Conclusions

This case highlights the importance of routine blood pressure measurement in the pediatric population as a key tool for the early detection of silent conditions such as UPJO. The insidious nature of pediatric hypertension requires clinical vigilance and a thorough diagnostic approach to intervene before irreversible organ damage occurs.

Rigorous monitoring, combined with multidisciplinary collaboration and active family engagement, is crucial for a favorable clinical outcome. Early investment in preventive strategies and timely diagnosis is essential not only for preserving renal function but also for safeguarding the long-term cardiovascular health of pediatric patients.
